# Field-based assessments of the seasonality of *Culex* *pipiens* sensu lato in England: an important enzootic vector of Usutu and West Nile viruses

**DOI:** 10.1186/s13071-024-06143-6

**Published:** 2024-02-11

**Authors:** Nicola Seechurn, M. Trent Herdman, Arturo Hernandez-Colina, Alexander G. C. Vaux, Colin Johnston, Morgan Berrell, Javier Lopez, Lindsay Eckley, Merit Gonzalez-Olvera, Lisa Gillespie, Paul Pearce Kelly, Matthew Baylis, Jolyon M. Medlock

**Affiliations:** 1 Institute of Infection, Veterinary and Ecological Sciences, University of Liverpool, Liverpool, UK; 2 Medical Entomology and Zoonoses Ecology Group, UK Health Security Agency, Porton Down, Salisbury, SP4 0JG UK; 3 UK Field Epidemiology Training Programme Field Service, South East and London, UK Health Security Agency, London, UK; 4 North of England Zoological Society (Chester Zoo), Caughall Road, Chester, CH2 1LH UK; 5 Twycross Zoo, East Midland Zoological Society, Burton Road, Atherstone, CV9 3PX UK; 6 Zoological Society of London, Outer Circle, Regent’s Park, London, NW1 4RY UK

**Keywords:** *Culex* spp, *Culexpipiens* s.l., *Culextorrentium*, Zoo, Disease ecology

## Abstract

**Background:**

Usutu virus (USUV), which is closely related to West Nile virus (WNV), sharing a similar ecology and transmission cycle, was first reported in the UK in the southeast of England in 2020. Both USUV and WNV are emerging zoonotic viruses hosted by wild birds. The 2020 finding of USUV in England raised awareness of this virus and highlighted the importance of understanding the seasonality of *Culex* *pipiens* sensu lato (*Cx. pipiens* s.l.), the main enzootic vector of these viruses. Zoos are prime locations for trapping mosquitoes because of their infrastructure, security, and range of vertebrate hosts and aquatic habitats.

**Methods:**

Three independent zoo-based case studies at four locations that cover the seasonality of *Cx.* *pipiens* s.l. in England were undertaken: (i) London Zoo (Zoological Society London [ZSL]) and surrounding areas, London; (ii) Chester Zoo (Cheshire); (ii) Twycross Zoo (Leicestershire); and (iv) Flamingo Land (zoo; North Yorkshire). Various adult mosquito traps were used to catch adult *Cx.* *pipiens* s.l. across seasons.

**Results:**

High yields of *Cx.* *pipiens* s.l./*Culex torrentium* were observed in Biogents-Mosquitaire and Center for Disease Control and Prevention Gravid traps in all studies where these traps were used. Mosquito counts varied between sites and between years. Observations of adult *Cx.* *pipiens* s.l./*Cx. torrentium* abundance and modelling studies demonstrated peak adult abundance between late July and early August, with active adult female *Cx.* *pipiens* s.l./*Cx. torrentium* populations between May and September.

**Conclusions:**

The information collated in this study illustrates the value of multiple mosquito monitoring approaches in zoos to describe the seasonality of this UK vector across multiple sites in England and provides a framework that can be used for ongoing and future surveillance programmes and disease risk management strategies.

**Graphical Abstract:**

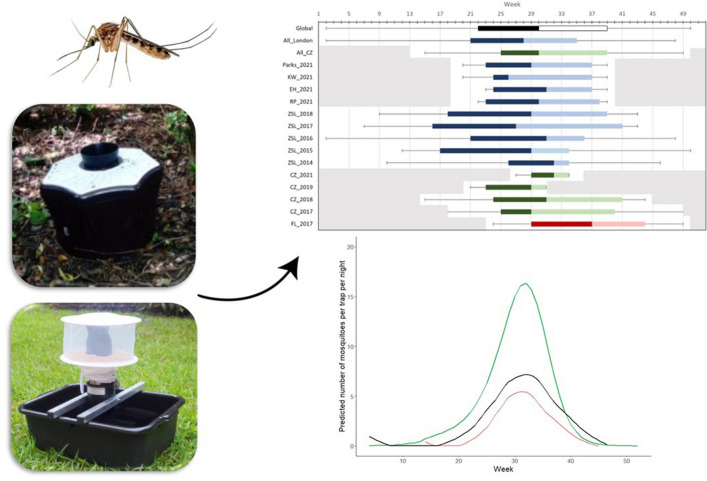

**Supplementary Information:**

The online version contains supplementary material available at 10.1186/s13071-024-06143-6.

## Background

Usutu virus (USUV) is an emerging zoonotic flavivirus that is phylogenetically related to West Nile virus (WNV), with a distribution across Africa and Europe; it also represents a potential health threat to humans and animals [1–3]. USUV cycles enzootically between birds and bird-biting mosquitoes. *Culex* spp. are the main vector of USUV, with *Culex* *pipiens* sensu lato (*Cx. pipiens* s.l.) Linnaeus, 1758, being the predominant enzootic vector in Europe [4]. Migratory birds, which are believed to have introduced USUV from Africa to Europe, do not show high levels of observable disease or mortality [2, 3, 5]. Since this introduction, passerine bird species, such as the Eurasian blackbird (*Turdus* *merula*), have disseminated USUV within Europe. Unlike migratory birds, infected blackbirds show a high mortality [6, 7]. Death has also occurred in Great grey owls (*Strix* *nebulosa)* in a zoo in Vienna following USUV infection [8, 9]. In 2020, the UK reported its first evidence of USUV transmission, with detection of this virus from infected birds and *Culex* *pipiens* s.l. mosquitoes in London, prompting enhanced field studies on this vector [10, 11].

In common with other members of the Japanese Encephalitis virus (JEV) serocomplex, USUV and WNV infect humans as incidental, dead-end hosts, via bridge vectors that are both bird-biting and human-biting [12]. The spectrum of human disease ranges from asymptomatic seroconversion to mild febrile illness to rare cases of meningoencephalitis or mononeuropathy, with severe disease more commonly occurring in immunocompromised individuals [13]. Under-ascertainment of human disease is likely, given the frequency of subclinical infection, limited availability of diagnostic testing and potential misattribution of infections due to high serological cross-reactivity with other flaviviruses [14].

Zoological gardens (here referred to as ‘zoos’) are important sites of entomological surveillance and research [15–17]. As unique environments in which native and non-native species of animals coexist, they can facilitate interactions among hosts and pathogens of potential importance to animal and human health [8, 18]. The relevance of mosquito ecology in zoos to human and animal health, and the potential role of zoos in sentinel surveillance, is exemplified by the outbreak of WNV in the Bronx Zoo/Wildlife Conservation Park [19].

Entomological research and surveillance help identify and mitigate threats to human and animal health from emerging vector-borne diseases by driving risk assessment programmes and action by public, animal and environmental health institutions [20]. Climate change makes vigilance and preparedness even more important, as the ecology of vectors and migratory hosts is changing [21]. Assessing the health risks of USUV requires entomological surveillance and research focused on its principal vector, *Cx.* *pipiens* s.l., which is widespread throughout the UK in urban and rural habitats [22, 23]. However, the seasonality and responsiveness of this mosquito to different trap designs and locations has not been systematically assessed in this context.

In the UK, *Culex* *pipiens* s.l. comprises two biotypes: the ornithophilic *Culex* *pipiens* typical form and the mammalophilic *molestus* form, as well as hybrids of the two [24]. A separate species, *Culex* *torrentium* Martini, 1925, is also present although usually rarely recorded [25]. This latter species is morphologically indistinguishable from *Culex* *pipiens* s.l. and is also an arbovirus vector [26, 27]. In most studies that rely on morphological identification, individuals described as ‘*Culex* *pipiens* s.l.’ could be any of these types.

In this paper we present data collated from routine and enhanced surveillance for *Cx.* *pipiens* s.l*./Cx. torrentium* conducted in and around zoological gardens in England. We describe the seasonality and behaviour of *Cx.* *pipiens* s.l./*Cx. torrentium* in these unique ecological environments. Bringing these descriptions together enables us to refine surveillance techniques and better understand the diversity, abundance and seasonality of potential vectors of emerging infections.

## Methods

Entomological surveillance was conducted at the following sites: (i) London Zoo (Zoological Society London [ZSL]) and surrounding parks, including Regent’s Park and Hampstead Heath, from 2014 to 2021; (ii) Chester Zoo, Cheshire (CZ), from 2017 to 2019 and 2021; (iii) Flamingo Land (zoo), North Yorkshire (FL), in 2017; and (iv) Twycross Zoo, Leicestershire (TZ), in 2021**.**

A variety of different traps were used at individual study sites throughout the sampling periods (Additional file 1: Table 1): (i) the BG-Mosquitaire mosquito trap (MQ; Biogents AG [BG], Regensburg, Germany) with BG-Sweetscent lactic acid attractant or BG-Lure lactic acid attractant (Biogents AG); (ii) the BG-Sentinel-2 mosquito trap (BGS; Biogents AG) with BG-Lure lactic acid attractant (Biogents AG); (iii) Centers for Disease Control and Prevention (CDC) Gravid traps (CDCG; John W. Hock Co., Gainesville, FL, USA) with water or hay infusion; (iv) Mosquito Magnet Executive trap (MM; Woodstream Corp., Lancaster, PA USA) with R-Octenol (Woodstream Corp.); (v) resting boxes (RB; wooden boxes approximately 50 cm^3^, open on one side, painted red inside and black outside; UK Health Security Agency [UKHSA] and University of Liverpool); and (vi) CDC Backpack Aspirators model 1412 (BA; John W. Hock Co.).

**Table 1:** Mosquito species collected at each study site in specific years

Genus	Species	Location of collection site and year of collection							Total
		ZSL	CZ				FL	TZ	
		2021	2017	2018	2019	2021	2017	2021	
*Anopheles*	*claviger*	6	2	4	2	2	0	0	16
	*plumbeus*	220	2	1	1	0	0	0	224
	*maculipennis* s.l	0	11	28	15	0	0	0	54
	Unknown	0	3	1	0	0	0	0	4
*Aedes*	*annulipes*	0	0	0	2	0	0	0	2
	*detritus*	0	0	0	1	0	0	0	1
	*vexans*	0	0	0	2	0	0	0	2
	*geniculatus*	3	0	0	0	0	0	0	3
*Coquillettidia*	*richiardii*	20	5	2	15	1	0	0	43
*Culex*	*pipiens* s.l./*torrentium*^a^	2896	0	2278	2519	1390	0	1310	10,393
	*pipiens* s.l^b^	NA	6163	NA	NA	NA	1124	0	7287
	*torrentium*^b^	NA	48	NA	NA	NA	60	NA	108
	Unknown	0	927	126	304	0	90	NA	1447
*Culiseta*	*annulata*	134	276	128	71	29	31	21	690
	*morsitans*	0	3	12	4	0	0	0	19
	Unknown	0	21	13	27	0	1	0	62
Unknown		3	477	359	328	0	271	0	1438

Numbers in table are the number of mosquitoes of each species caught at each site in a specific collection year.

*CZ* Chester Zoo, *Fl* Flamingo Land, *NA* not applicable,* s.l.* sensu lato, *TZ* Twycross Zoo, *ZSL* Zoological Society of London

^a^Differentiation between *Cx.* *pipiens* and *Cx.* *torrentium* was not possible as only morphological identification was undertaken

^b^Molecular methods were used to identify *Cx.* *pipiens* s.l. and *Cx.* *torrentium*

A hay infusion was prepared and maintained in each CDCG trap, following the protocol developed by Reiter [28], with fresh hay infusion medium provided once weekly. When fresh water was used in the CDCG traps, the water was changed upon observation of larvae or excessive debris accumulation (approximately once monthly). RB traps were inspected once a week, and all mosquitoes in the traps were collected using either a mouth aspirator or BA. The lure for MM traps was changed once monthly. BG-Sweetscent was replaced once monthly and the BG-lure was replaced every 5 months.

Traps at ZSL and the surrounding parks were continuously operated, with the catch bags changed weekly. On the occasions when the catch bag was left in place for more than 1 week, the catch was averaged over the number of weeks since the last exchange. At CZ and FL in 2017 and CZ in 2018 and 2021, MQ traps were constantly operated with alternating 1-day and 6-day catches. CDCG traps were operated once a week for a 1-day collection, running at the same time as the 1-day catch of the MQ traps. In 2019, MQ traps were operated for 5 days, followed by 2 days, and CDCG traps were operated for 2 consecutive days. At TZ (2021), all traps were operated 1 day per week. The traps used for each zoo are shown in Additional file 1: Table A1.

Female mosquitoes were identified morphologically to species level using appropriate keys on a cold plate or chill table [29, 30]. In addition to morphological identification, molecular identification was also undertaken to differentiate *Cx.* *pipiens* s.l. and *Cx.* *torrentium* for those specimens collected at CZ and FL in 2017 [31]. DNA was extracted from pools of up to 10 legs of individual *Cx.* *pipiens* s.l./*Cx. torrentium* using the OMEGA Bio-Tek E.Z.N.A® Tissue DNA Kit (Omega Bio-Tek Inc., Norcross, GA, USA) If pools containing *Cx.* *pipiens* s.l. and *Cx.* *torrentium* were identified, individual mosquitoes were rescreened. When DNA methods were not used to distinguish between *Cx.* *pipiens* biotypes or *Cx.* *torrentium*, the specimens are referred to here as *Cx.* *pipiens* s.l./*torrentium*.

### Time-series analysis for mosquito seasonality

Weekly estimates of ZSL MQ-trapped female *Cx.* *pipiens* s.l./*torrentium* per trap-week all year round from 2014 to 2018 were imported into the Stata version 14 software package [32] for modelling of periodicity and time-series analysis. Zero-inflated negative binomial regression was undertaken for the time-series analysis, based on high variance and an expected winter season with no counts for most observations. The winter season was defined by a variable that was coded zero for weeks in which no *Cx.* *pipiens* s.l.*/torrentium* were caught or for catches separated from the main season of abundance by more than 2 consecutive weeks with no catch. A 52-week seasonal period was assumed a priori and included with the equations cos(2*pi*date/52) and sin(2*pi*date/52) as independent variables. Additional periodicity within the time series was sought by inspecting residuals, and was included when the fit of the model increased, as determined by a likelihood ratio test. The model was validated qualitatively by comparison to seasonality observed at all ZSL study sites in 2021. For trap-catch data where excess zeros were not observed (CZ from 2017 to 2019 and 2021), a negative binomial model was generated in RStudio using the MASS and pscl packages [33, 34].

### Analysis of landscape variables

In 2017 and 2018, landscape variables around the sampling areas at CZ were recorded, including vegetation, proximity to water sources, animal enclosures and resting areas excluding vegetation. Daily regional temperature, relative humidity, wind speed and precipitation were obtained from the OGIMET Weather Information Service (www.ogimet.com) of the closest weather stations to the zoos using the R package Climate. Tingtag© dataloggers (Gemini Data Loggers Ltd., Chichester, West Sussex, UK) were placed next to the MQ traps and were programmed to record every hour.

Untransformed mosquito collection data were analysed in relation to weather and landscape variables (i.e. vegetation, distance to water bodies, animal exhibits, resting areas, temperature, humidity and rainfall) using a generalised linear model (GLM) with a negative binomial distribution. Mosquito collections were separated to analyse host-seeking and ovipositing preferences.

## Results

### Overall catch data

At CZ, year-round surveillance using MQ traps from 2014 to 2018 principally yielded female *Cx.* *pipiens* s.l./*torrentium*. Over this period, the number, sex and species of mosquitoes trapped included 7045 female *Cx.* *pipiens* s.l./*torrentium*, 135 female *Culiseta* *annulata* Schrank, 1776 and one female *An.* *Plumbeus* Stephens, 1828, as well as 504 male *Cx.* *pipiens* s.l./*torrentium* and eight male *Cs.* *annulata*. At CZ, a total of 16,607 *Cx.* *pipiens* s.l./*torrentium* were collected across all sampling years. At FL, 1124 adult *Cx.* *pipiens* s.l./*torrentium* mosquitoes were trapped in 2017, and at TZ, 1310 *Cx.* *pipiens* s.l.*/torrentium* were collected in 2021. In total, 13 different mosquito species or species complexes were collected across all sites (see Table 1).

**Fig. 1:**
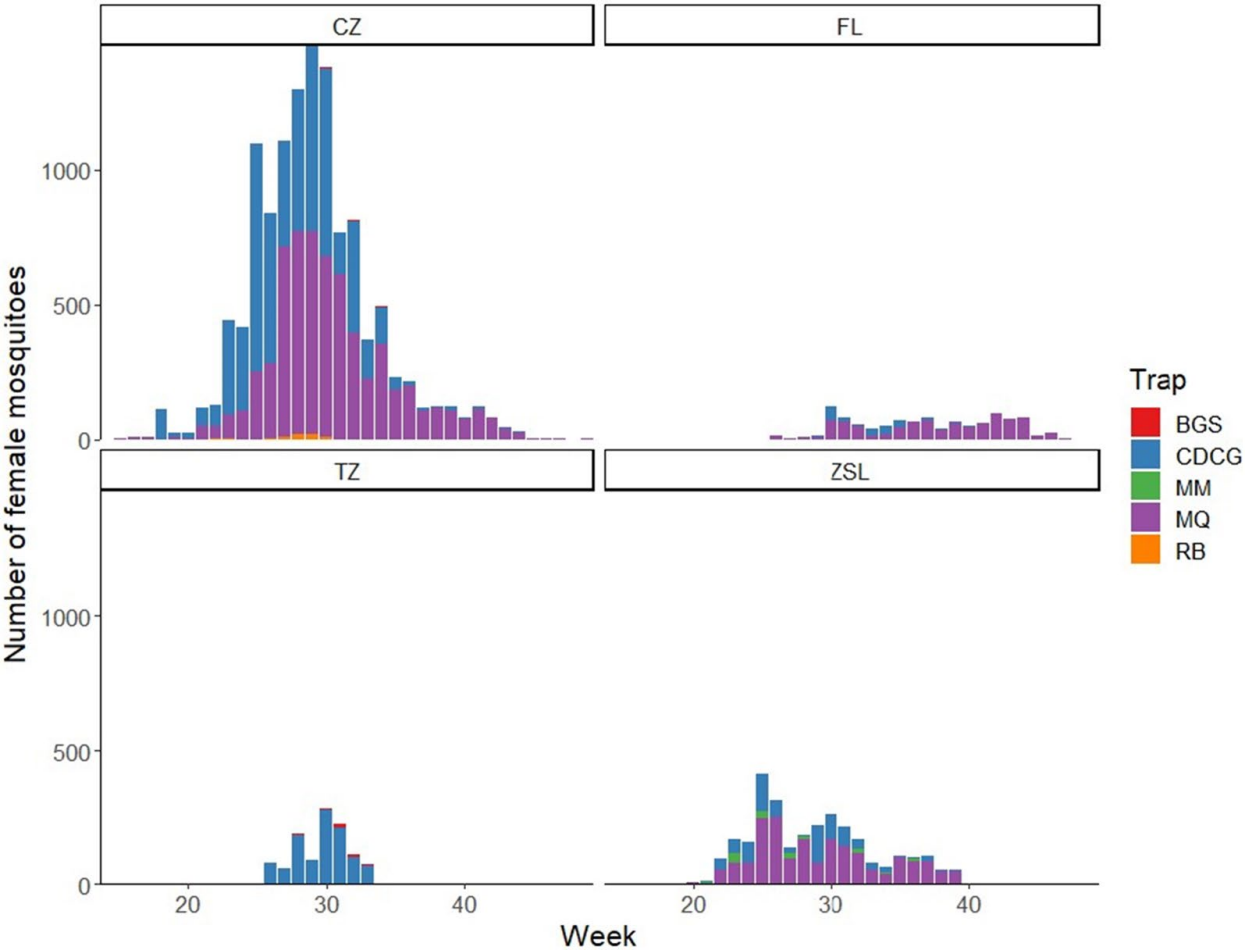
Trap-catch data on adult female *Culex* *pipiens* sensu lato/*Culex torrentium* by trap type. Data for ZSL include data from all London Park sites, week 20 to week 39 (early May to late September), 2021. Data at CZ are from week 18 to week 49, 2017–2019 and 2021. Data at FL are from week 24 to week 50, 2017. Data from TZ are from week 26 to week 34, 2021. Trapping sites: CZ, Chester Zoo; FL, Flamingo Land; TZ, Twycross Zoo; ZSL, Zoological Society of London. Collection traps: CDCG, CDC Gravid trap; MM, Mosquito Magnet trap (operating in alternate weeks at each site for ZSL); MQ, Mosquitaire trap; RB, resting box

In our dataset, the majority of *Cx.* *pipiens* s.l./*torrentium* (53.66%; *n* = 9245) were collected in MQ traps, with the second most *Cx.* *pipiens* s.l./*torrentium* mosquitoes (44.92%; *n* = 7739) collected in CDCG traps. The remaining 1.42% of *Cx.* *pipiens* s.l./*torrentium* mosquitoes were collected in a mix of MM, RB and BGS traps (Fig. 1). Trap-catch data at ZSL and CZ are from 2021 and from 2017 to 2019 and 2021, respectively.

### Analysis of landscape variables on trap-catch

The negative binomial GLM showed that temperature, dense vegetation and proximity to water bodies and animal exhibits were positively associated with mosquito count at CZ and FL in 2017 regardless of mosquito behaviour (host-seeking or oviposition site-seeking). The strongest association in all cases was with temperature (for details, see Additional file 2: Table A2).

### Count data modelling

**Fig. 2:**
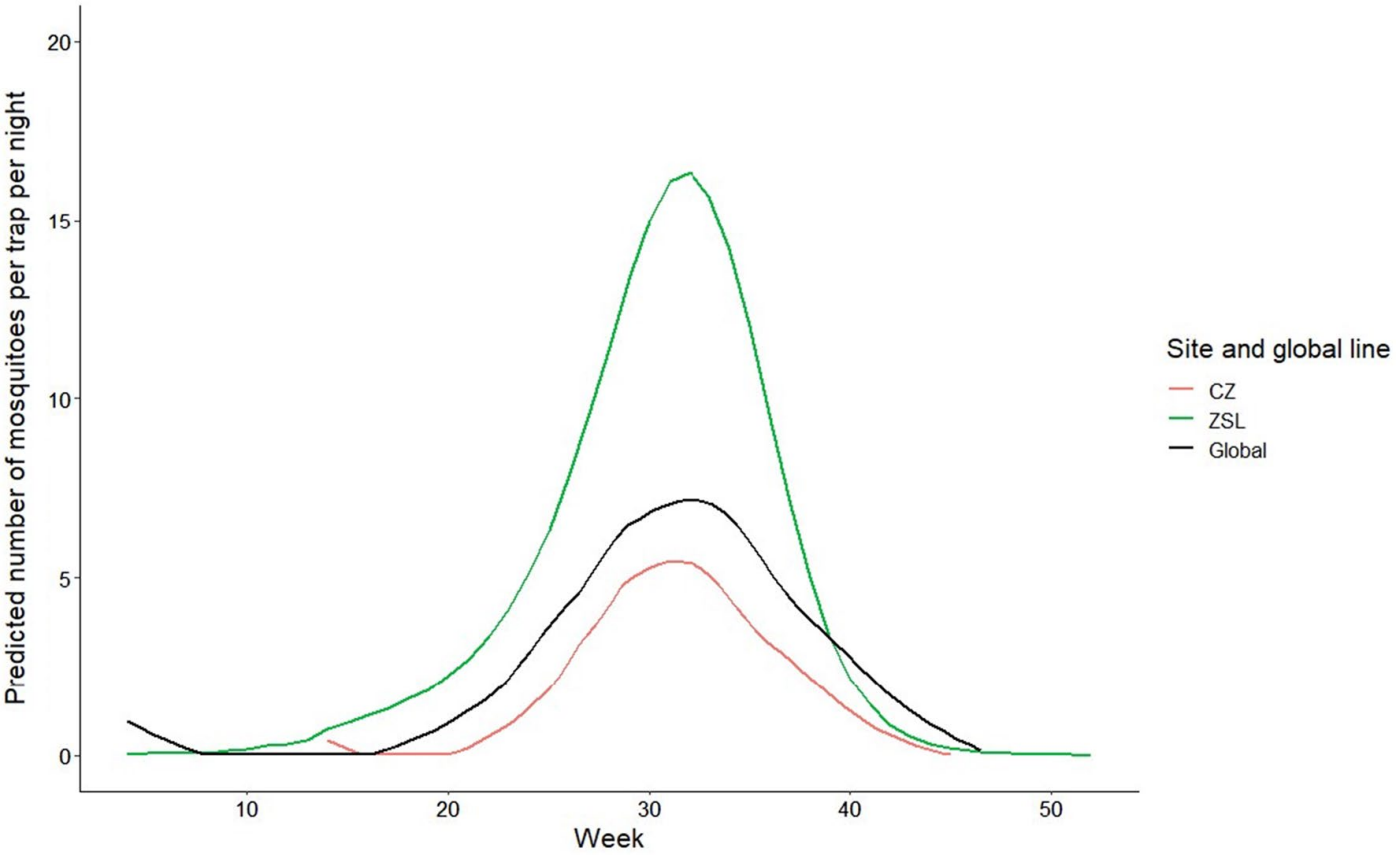
Seasonal change in trap-catch of *Cx.* *pipiens* s.l./*torrentium* at ZSL from 2014 to 2018 and at CZ from 2017 to 2019 and in 2021. Female mosquitoes were caught using Biogents-Mosquitaire (MQ) traps. Data generated for ZSL were obtained using a zero-inflation negative binomial model derived from the observed yield from 2014 to 2018, while data from CZ were derived from a negative binomial model as excess zeros were not observed in the CZ dataset. CZ, Chester Zoo; ZSL, Zoological Society of London

Longitudinal data from ZSL collected between 2014 and 2018 were used to assess the change in mosquito trap-catch over time. Over the five collection seasons at ZSL, peak catches ranged from 17 to 47 female *Cx.* *pipiens* s.l./*torrentium* per trap-night, with a median of 32 (early August), across week 29 to week 35 (mid- July to late August) in each year. The female *Cx.* *pipiens* s.l./*torrentium* catches over this period for ZSL and CZ are shown in Fig. 2. The time-series analysis model was improved by inclusion of periodicities of 52 and 26 weeks and zero-inflation in the off-season (all *P* < 0.001), to generate a prediction curve peaking at week 32 (early August) and > 1 mosquito per trap-night from week 16 to week 41 (mid-April to early/mid-October). The negative binomial model additionally generated a curve peaking at a similar time at ZSL; however, the peak in trap-catch at CZ was more stepped than the peak observed at ZSL. Raw data from ZSL from 2014 to 2018 and from CZ from 2017 to 2019 and 2021 were used to generate the prediction curves.

### Yearly distribution of female* Cx. pipiens* s.l./*torrentium*

**Fig. 3:**
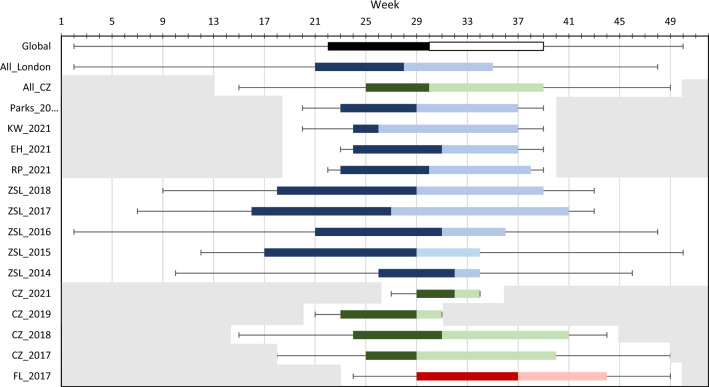
Temporal distribution in the trap-catch of host-seeking female *Cx.* *pipiens* s.l./*torrentium* at all study sites across all years for which data are available (2014–2021). Whiskers show the range from the first to last week in which *Cx.* *pipiens* s.l./*torrentium* mosquitoes were caught. Boxes show the cumulative yield of 5%, 50% and 95%, with 5% cumulative yield represented by the left side of the darker box; 50% cumulative yield, by the junction between dark and light boxes; and 95% cumulative yield, by the right side of the light box. Collection sites are indicted by colour-coded boxes, with blue boxes representing data from ZSL and surrounding parks; green boxes, data from CZ; and the red box, data from FL. Grey shading represents the time when traps were not running. CZ, Chester Zoo; EH, East Heath (Hampstead Heath);FL, Flamingo Land; KW, Kenwood Yard (Hampstead Heath); RP, Regent’s Park; ZSL, Zoological Society of London

Trap-catch data on host-seeking female *Cx.* *pipiens* s.l./*torrentium* across all sites sampled are shown in Fig. 3. While year-round surveillance data at ZSL between 2014 and 2018 demonstrates that mosquitoes are sporadically trapped throughout the year, there is a clear overall season of greater abundance from May to September. Nearly 90% of the total yield across all London sites was caught from week 20 to week 37 (mid-May to mid-September). While the peak yield was variable from site to site and from year to year, the median time point at which 50% of the annual yield was reached was week 30 (late July) (see the Global line in Fig. 2), which was 2 weeks earlier than at CZ.

### Center for Disease Control and Prevention Gravid traps

Collections from the CDCG traps at CZ and TZ produced multiple, asynchronous peaks in adult *Cx.* *pipiens* s.l./*torrentium* catches. *Culex* *pipiens* s.l./*torrentium* collections in CDCG traps at CZ produced three large catches separated by approximately 4-week intervals, namely at week 26 (late June), week 29 (mid-July) and week 32 (early August). Large catches of *Cx.* *pipiens* s.l./*torrentium* in CDCG traps at TZ occurred on week 28 (mid-July) and week 31 (early August). The large catches of *Cx.* *pipiens* s.l./*torrentium* at TZ appeared approximately 1 week earlier than the large catches of *Cx.* *pipiens* sl./*torrentium* at CZ or, alternatively, 3 weeks following the large catches at CZ.

## Discussion

This description of *Cx.* *pipiens* s.l./*torrentium* distribution across three independent studies covering 7 years and 4 locations in England provides an essential characterisation of the principal UK vector of USUV and WNV. While the methodologies used in the present study varied across studies, high yields of *Cx.* *pipiens* s.l./*torrentium*, especially from the MQ and CDC Gravid traps, were found in all studies where these traps were used. Peak catch varied from year to year, and from site to site within a single year in all studies but peak catches were obtained between weeks 28 and 35, equivalent to mid-July to late August. Median abundance, a measure of 50% of the annual catch, occurred between July and early August in all surveys, which corresponds to the modelled peak of between late July and early August based on earlier ZSL and CZ observations. In addition, we identified the maximum effective trapping season to occur between mid-April and early October, and found evidence that temperature predicts the abundance of *Cx.* *pipiens* s.l. in the short-term.

Our findings suggest that the MQ and CDC Gravid traps are useful for *Culex* surveillance. Both anticipated yield and operational practicalities of all traps should be considered in the design of future surveillance. The MQ trap is particularly physically robust, can be powered by mains electricity or be modified to use battery packs or solar power and can be used along with the CDC Gravid and BGS traps. In terms of trap maintenance, the MQ and BGS traps require that an operator exchange catch nets and maintain the lure on a regular basis, and water reserves and battery power renewal are essential for the CDC Gravid trap. However, these three traps require less maintenance to operate than the MM traps and RBs, and are associated with fewer logistical issues (e.g. use of flammable propane in the MM trap).

The MQ, CDC Gravid and BGS traps all attract *Cx.* *pipiens* s.l./*torrentium* with the use of lure only, and do not require CO_2_. A variety of lures were used in the studies reported here, including CO_2_ (sourced as dry ice and propane), BG-Sweetscent, BG-Lure and octenol. These lures are designed to attract host-seeking female mosquitoes and, therefore, traps utilising these lures will sample this large proportion in this nulliparous life stage of the adult mosquito. CDC Gravid traps attract females seeking ovipositing sites, which are important in terms of virological surveillance as these gravid females have already undergone one gonotrophic cycle and, therefore, theoretically are more likely to be positive for virus. The practicality of implementing the various types of traps described here in the field should also be considered. As such, using a variety of mosquito traps provides information on the different adult life stages of *Cx.* *pipiens* s.l./*torrentium* which can be sampled. Given the long season and variable peaks observed using different trap types for *Cx.* *pipiens* s.l.*,* choosing effective traps that can be easily operated by stakeholders over a sufficient period of time is an important consideration. There is a dearth of literature on the seasonality of *Cx.* *pipiens* s.l. in the UK and, to our knowledge, no published data are available which compare seasonality between geographic locations. A study by Ewing et al. [35] used field collection abundance data on eggs, larvae, pupae and adult stages to model seasonality in the UK, but the data were limited by sampling at only one location (Wallingford, Oxfordshire) and a limited number of immature and adult sampling points. Nevertheless, these authors demonstrated peak adult abundance at the end of July. *Culex* *pipiens* s.l. seasonality has been modelled in continental Europe using observations from France, Greece, Italy and Serbia [36], with a similar abundance pattern as reported in the present study.

While our collation of data from multiple study sites provided robust data on the seasonality of *Cx.* *pipiens* s.l./*torrentium* in England, the differing methodologies used limits direct comparison and full synthesis of data across all studies. MM traps were only used at one location, which biases sampling of variations in general mosquito species at these sites toward mammal-biting species, given the use of a lure in MM traps that is designed to attract mammal-biting species. Shorter trapping seasons occurred in some series, which limits extrapolation beyond these months. The study sites chosen may not be representative of the UK as a whole, given that sites were selected to be close to captive bird species or large areas of park and heath land. While these limitations introduce a degree of sampling bias, they reflect the importance of surveillance for mammal-biting species in proximity to potential bird hosts for detection of certain arboviruses. Furthermore, enhanced surveillance in and around ZSL in 2021 provided maximal insight into the area most affected by USUV.

At some sites, species identification was limited to morphological identification, which does not distinguish between *Cx.* *pipiens* s.l. and *Cx.* *torrentium*. Therefore, the true genetic diversity of *Cx.* *pipiens* s.l. may have been missed. Where molecular identification of *Cx.* *pipiens* and *Cx.* *torrentium* was undertaken, only 0.6% of the *Cx.* *pipiens* s.l./*torrentium* were identified as *Cx.* *torrentium*, which suggests that *Cx.* *pipiens* s.l. was the predominant species collected. However, further research aimed at improving our understanding of the relative distribution and abundance of these two species is required. None of the studies reported here distinguished between the biotypes of *Cx.* *pipiens* s.l.*,* which would be a valuable consideration for future studies. Distinction between the bird-biting *Cx.* *pipiens* *pipiens*, the human-biting *Cx.* *pipiens* *molestus* and hybrid populations is a crucial epidemiological factor that should be considered when conducting disease risk assessments and pathogens management programmes between birds and people.

Seasonality has a direct impact on the transmission season of arboviruses such as USUV and WNV [37]. Therefore, defining and understanding the factors that affect *Cx.* *pipiens* s.l. seasonality is imperative to defining the transmission of emerging vector borne diseases in the UK. With the added impact of climate change causing more extreme heat events, the impact of climate change on the vectorial capacity of *Cx.* *pipiens* s.l. and, consequently, the potential USUV and WNV transmission season, needs to be better characterised. A time-series model could include other variables that may influence seasonality, such as local temperature, rainfall or humidity. However, using such variables for predictive models has a limited benefit as these variables will vary unpredictably from year to year.

We observed that weather and landscape features significantly affected *Cx.* *pipiens* s.l. abundance in relatively small areas, with the regional temperature being a possible main driver of mosquito activity, followed by dense vegetation and proximity to water sources and animal exhibits as other potential drivers. Therefore, the local environment should be considered in active surveillance studies of mosquitoes in areas of interest.

## Conclusions

The results from this study provide a framework for enhanced surveillance of *Cx.* *pipiens* s.l. in response to detection of mosquito-borne diseases such as USUV and WNV. The range and intensity of trapping was expanded to appropriate sites to determine the abundance of the vector and allow specimens to be collected for viral detection. To guide such efforts, we have described the anticipated seasonality of this vector across a large geographic area in England.

### Supplementary Information

**Additional file 1: Table A1.** Traps used in all studies. ^a^Morphological identification was undertaken using morphological keys. ^b^Molecular identification of *Culex* *pipiens* s.l. from *Culex* *torrentium* was undertaken using the protocol specified by Hesson et al. [31]. ^c^On occasions when the catch bag was left in place for > 1 week, the catch was averaged over the number of weeks since the last exchange. ^d^Specimens were stored at - 20 ˚C prior to morphological identification and then stored at - 80 ˚C following identification for long-term storage. ^e^Propane was used as a CO_2_ source. ^f^ In weeks 48 and 49 (late November to early December), the inside of buildings, sheds and animal enclosures near sampling areas were aspirated with a BA in FL and CZ in 2017, respectively. ^g^ The inside of buildings, shed and animal enclosures were aspirated in week 5 (late January), 2018. ^h^Dry ice was used as a carbon dioxide source. ^i^Vegetation surrounding RBs were aspirated for 5 min. BA, Backpack Aspirator; BG, Biogents Germany; BGS, BG-Sentinel-2 trap; CDCG, Center for Disease Control and Prevention Gravid trap; MM, Mosquito Magnet Executive Trap; MQ, BG-Mosquitaire trap; RB, resting box; ZSL, Zoological Society of London.

**Additional file 2: Table A2.** Significant variables in generalised linear models (GLMs) of weather and landscape variables on the catch size of *Cx* *pipiens* s.l./*torrentium* mosquitoes. Arrows pointing upwards indicate a positive influence, and arrows pointing downwards, a negative one on mosquito catch size. *P*-value provided in brackets. Asterisks indicate significant difference at *P < 0.05, **P < 0.01 and *** *P* < 0.001.

## Data Availability

All data relevant for this study are contained in this published article and the additional files. The datasets generated and/or analysed during the current study are available from the corresponding author upon reasonable request

## Abbreviations

BA: CDC Backpack Aspirator
BG: Biogents
BGS: Biogents-Sentinel-2
CDC: Centers of Disease Control and Prevention
CDCG: CDC Gravid trap
CZ: Chester Zoo
FL: Flamingo Land
GLM: Generalised linear model
MM: Mosquito Magnet Executive (insect trap)
MQ: Mosquitaire (mosquito trap)
RB: Resting box
TZ: Twycross Zoo
USUV: Usutu virus
WNV: West Nile virus
ZINB: Zero-inflated negative binomial
ZSL: Zoological Society of London
