# Supplementary material for: Field-based assessments of the seasonality of Culex pipiens sensu lato in England: an important enzootic vector of Usutu and West Nile viruses
Source: Parasit Vectors. 2024 Feb 11;17:61. doi: 10.1186/s13071-024-06143-6 (PMC10859028; doi:10.1186/s13071-024-06143-6)
Supplement: Supplementary file 1 — Additional file 1: Table A1. Traps used in all studies. aMorphological identification was undertaken using morphological keys. bMolecular identification of Culex pipiens s.l. from Culex torrentium was undertaken using the protocol specified by Hesson et al. [31]. cOn occasions when the catch bag was left in place for > 1 week, the catch was averaged over the number of weeks since the last exchange. dSpecimens were stored at - 20 ˚C prior to morphological identification and then stored at - 80 ˚C following identification for long-term storage. ePropane was used as a CO2 source. f In weeks 48 and 49 (late November to early December), the inside of buildings, sheds and animal enclosures near sampling areas were aspirated with a BA in FL and CZ in 2017, respectively. g The inside of buildings, shed and animal enclosures were aspirated in week 5 (late January), 2018. hDry ice was used as a carbon dioxide source. iVegetation surrounding RBs were aspirated for 5 min. BA, Backpack Aspirator; BG, Biogents Germany; BGS, BG-Sentinel-2 trap; CDCG, Center for Disease Control and Prevention Gravid trap; MM, Mosquito Magnet Executive Trap; MQ, BG-Mosquitaire trap; RB, resting box; ZSL, Zoological Society of London. [file 13071_2024_6143_MOESM1_ESM.pdf]

**Table A1: Trap types used in all studies.** [a]Morphological identification was undertaken using morphological keys. [b]Molecular identification of *Culex pipiens* sl. from *Culex torrentium* was undertaken using the protocol specified by Hesson *et al* [31]. [c]On occasions when the catch bag was left in place for more than one week, the catch was averaged over the number of weeks since the last exchange. [d]Specimens stored at -20 ˚C prior to morphological identification and were then stored at -80 ˚C following this for long-term storage. [e]Propane was used as a carbon dioxide source. [f] In Week 48 and 49 (late November to early December), the inside of buildings, sheds, and animal enclosures near sampling areas were aspirated with a BA in FL and CZ in 2017, respectively. [g] The inside of buildings, shed and animal enclosures were aspirated in Week 5 (late January), 2018. [h]Dry ice was used as a carbon dioxide source. [i]Vegetation surrounding RBs were aspirated for five minutes. BA: Backpack Aspirator. BG: Biogents Germany. BGS: BG-Sentinel-2 trap. CDCG: Centre for Disease Control and Prevention Gravid trap. MM: Mosquito Magnet Executive Trap. MQ: BG-Mosquitaire trap. RB: resting box. ZSL: Zoological Society of London.

| Location | Year | Trap type | Attractant | Trap operation | | Net replacement | Mosquito identification | | Storage | |
|---|---|---|---|---|---|---|---|---|---|---|
| | | | | Start | End | | [a]Morphological | [b]Molecular | -20 ˚C | -80 ˚C |
| ZSL London Zoo | 2014 - 2018 | MQ | BG-Sweetscent | Week 1 | Week 52 | [c]Once a week | ✓ | ✗ | [d]✓ | [d]✓ |
| ZSL London Zoo, Regent's Park and Hampstead Heath | 2021 | MQ | BG-Sweetscent | Week 19 | Week 42 | Once a week | ✓ | ✗ | [d]✓ | [d]✓ |
| | | [e]MM | R-Octenol | | | | | | | |
| | | RB | None | | | | | | | |
| | | CDCG | Standing water | | | | | | | |

| | | | | | | | | | | |
|---|---|---|---|---|---|---|---|---|---|---|
| Chester Zoo & Flamingo Land | 2017 – 2018 | MQ | BG-Sweetscent | [f]Week 18 (2017, CZ) [g]Week 14 (2018, CZ) [f]Week 23 (2017, FL) | [f]Week 49 (2017, CZ) [g]Week 45 (2018, CZ) [f]Week 50 (2017, FL) | Twice a week | ✓ | ✓ | ✓ | ✗ |
| | | CDCG | Hay infusion | | | Once a week | | | | |
| Chester Zoo | 2019 | [h]MQ | BG-Sweetscent | Week 21 | Week 31 | Twice a week | ✓ | ✗ | ✓ | ✗ |
| | | CDCG | Hay infusion | | | Once a week | | | | |
| | | RB | None | | | | | | | |
| | | [i]BA | None | | | | | | | |
| Chester Zoo | 2021 | MQ | BG-Lure | Week 25 | Week 35 | Twice a week | ✓ | ✗ | ✗ | ✓ |
| | | BGS | BG-Lure | | | Once a week | | | | |
| | | CDCG | Hay infusion | | | | | | | |
| | | RB | None | | | | | | | |
| | | [i]BA | None | | | | | | | |
| Twycross Zoo | 2021 | BGS | BG-Lure | Week 26 | Week 34 | Once a week | ✓ | ✗ | ✗ | ✓ |
| | | CDCG | Hay infusion | | | | | | | |
