# Supplementary material for: Field-based assessments of the seasonality of Culex pipiens sensu lato in England: an important enzootic vector of Usutu and West Nile viruses
Source: Parasit Vectors. 2024 Feb 11;17:61. doi: 10.1186/s13071-024-06143-6 (PMC10859028; doi:10.1186/s13071-024-06143-6)
Supplement: Supplementary file 2 — Additional file 2: Table A2. Significant variables in generalised linear models (GLMs) of weather and landscape variables on the catch size of Cx pipiens s.l./torrentium mosquitoes. Arrows pointing upwards indicate a positive influence, and arrows pointing downwards, a negative one on mosquito catch size. P-value provided in brackets. Asterisks indicate significant difference at *P < 0.05, **P < 0.01 and *** P < 0.001. [file 13071_2024_6143_MOESM2_ESM.pdf]

Table A2: Significant variables in generalised linear models (GLMs) of weather and landscape variables on the catch size of *Cx pipiens* s.l. mosquitoes. Arrows upwards indicate a positive influence and arrows downwards, a negative one on mosquito catch size. P value provided in brackets. *: p < 0.05; **: p <0.01; ***: p <0.001.

		**Host Search Behaviour**				**Ovi-positing Site Selection Behaviour**			
**Variable**		Overall	Chester Zoo 2017	Chester Zoo 2018	Flamingo Land	Overall	Chester Zoo 2017	Chester Zoo 2018	Flamingo Land
Regional Temperature		↑ (***)	↑ (***)	↑ (***)	↑ (***)	↑ (***)	↑ (***)	↑ (***)	↑ (*)
Humidity		↑			↑			↑	
Precipitation				↑ (*)				↓ (***)	
Vegetation	Dense	↑ (***)	↑ (***)	↑ (***)	↑ (**)	↑ (***)	↑ (***)		↑ (**)
	Medium						↑ (***)		
	Scarce					↓ (***)		↓ (***)	↓
Oviposition sites	Close	↑ (***)	↑ (***)	↑ (***)		↑ ***)		↑ (***)	
	Medium								
	Remote			↑ (**)				↑ (***)	
Resting Areas	Abundant			↓					
	Medium						↓ (*)		
	Rare	↓ (*)		↓ (***)	↓ (***)				
Zoo Animals	Close		↑	↑ (*)		↑ (***)		↑ (***)	↑ (***)
	Medium								
	Remote			↑		↑ (*)	↓ (**)	↑ (***)	
